# Virtual Reality Simulation Training for Cardiopulmonary Resuscitation After Cardiac Surgery: Face and Content Validity Study

**DOI:** 10.2196/30456

**Published:** 2022-03-02

**Authors:** Amir H Sadeghi, Jette J Peek, Samuel A Max, Liselot L Smit, Bryan G Martina, Rodney A Rosalia, Wouter Bakhuis, Ad JJC Bogers, Edris AF Mahtab

**Affiliations:** 1 Department of Cardiothoracic Surgery Erasmus University Medical Center Rotterdam Netherlands; 2 Educational Program Technical Medicine, Leiden University Medical Center Delft University of Technology Erasmus University Medical Center Rotterdam Leiden, Delft, Rotterdam Netherlands; 3 Medical Sciences Division University of Oxford Oxford United Kingdom; 4 Department of Clinical Research Zan Mitrev Clinic Skopje the Former Yugoslav Republic of Macedonia

**Keywords:** cardiac surgery, cardiopulmonary resuscitation, emergency resternotomy, virtual reality, simulation training, serious games, virtual reality simulation, digital health, medical training, virtual training

## Abstract

**Background:**

Cardiac arrest after cardiac surgery commonly has a reversible cause, where emergency resternotomy is often required for treatment, as recommended by international guidelines. We have developed a virtual reality (VR) simulation for training of cardiopulmonary resuscitation (CPR) and emergency resternotomy procedures after cardiac surgery, the Cardiopulmonary Resuscitation Virtual Reality Simulator (CPVR-sim). Two fictive clinical scenarios were used: one case of pulseless electrical activity (PEA) and a combined case of PEA and ventricular fibrillation. In this prospective study, we researched the face validity and content validity of the CPVR-sim.

**Objective:**

We designed a prospective study to assess the feasibility and to establish the face and content validity of two clinical scenarios (shockable and nonshockable cardiac arrest) of the CPVR-sim partly divided into a group of novices and experts in performing CPR and emergency resternotomies in patients after cardiac surgery.

**Methods:**

Clinicians (staff cardiothoracic surgeons, physicians, surgical residents, nurse practitioners, and medical students) participated in this study and performed two different scenarios, either PEA or combined PEA and ventricular fibrillation. All participants (N=41) performed a simulation and completed the questionnaire rating the simulator’s usefulness, satisfaction, ease of use, effectiveness, and immersiveness to assess face validity and content validity.

**Results:**

Responses toward face validity and content validity were predominantly positive in both groups. Most participants in the PEA scenario (n=26, 87%) felt actively involved in the simulation, and 23 (77%) participants felt in charge of the situation. The participants thought it was easy to learn how to interact with the software (n=24, 80%) and thought that the software responded adequately (n=21, 70%). All 15 (100%) expert participants preferred VR training as an addition to conventional training. Moreover, 13 (87%) of the expert participants would recommend VR training to other colleagues, and 14 (93%) of the expert participants thought the CPVR-sim was a useful method to train for infrequent post–cardiac surgery emergencies requiring CPR. Additionally, 10 (91%) of the participants thought it was easy to move in the VR environment, and that the CPVR-sim responded adequately in this scenario.

**Conclusions:**

We developed a proof-of-concept VR simulation for CPR training with two scenarios of a patient after cardiac surgery, which participants found was immersive and useful. By proving the face validity and content validity of the CPVR-sim, we present the first step toward a cardiothoracic surgery VR training platform.

## Introduction

Every year, around 2 million patients undergo cardiac surgery worldwide [1]. The incidence of cardiac arrest after cardiac surgery ranges between 0.7% to 8%, with a survival rate of approximately 50% [2-5]. This relatively high survival rate can be explained by a high incidence of reversible causes precipitating the arrest, such as ventricular fibrillation (VF; 25%-50%), cardiac tamponade, hypovolemia, and tension pneumothorax [2,4-6]. Notably, aside from VF, external massage is often ineffective in these cases because of reduced diastolic filling of the heart, resulting in inadequate tissue and brain perfusion [2]. In light of these findings, the Society of Thoracic Surgeons Taskforce on Resuscitation After Cardiac Surgery published an expert consensus in 2017 to provide guidelines for developing local protocols for cardiopulmonary resuscitation (CPR) after cardiac surgery [2]. As reported in the guidelines, early recognition of the clinical signs and symptoms is essential, indicating that emergency resternotomy is required [2,5]. The majority of postoperative cardiac surgery emergencies requiring CPR will involve reopening the sternum [2,5]. Several studies have shown that training and practicing based on a structured protocol improve the time to recognize the need for resternotomy and the time to reopen the thorax [2,7]. Early resternotomy reduces complications and improves outcomes for patients with cardiac tamponade, hypovolemia, or tension pneumothorax [2,7]. However, the paucity of cardiac arrest after cardiac surgery limits the possibilities of clinical training for clinicians [8]. CPR training allows clinical staff to acquire theoretical knowledge on the protocol, together with the ability to physically perform the steps described within the protocol [9]. This is commonly taught in instructor-led training sessions, requiring multiple team members and resources [2]. Moreover, these classroom sessions are currently restricted due to precautionary measures taken during the COVID-19 pandemic [10].

Simulation training enables training of multiple cases with unlimited practice (and possible errors) without compromising patient safety or the need for setting up training sessions [8]. Virtual reality (VR), with 360-degree scenarios, can recreate a fully immersive, interactive, and realistic scenario in which the user can repeatedly train without the need for other supplies or participants. Moreover, VR can be used in a multiuser setting, allowing different users to be present in the same scenario while physically distanced [11]. Multiple studies have shown that simulation training effectively improves knowledge, confidence, motivation, and satisfaction with the training versus standard training methodology [8,9,12,13].

Quantifying outcomes and the validity of simulations is a difficult task. It is essential that a VR simulator is valid in the sense that it resembles a realistic situation and reinforces the appropriate skills and knowledge [14]. This validity consists of several subtypes, including face validity and content validity. Face validity relates to the realism of a simulator, or in this case how well the simulation resembles real-world clinical practice [14,15]. This can be assessed informally by experts (referents) and nonexperts (novices/trainees) in the field [16-19]. Content validity judges the usefulness of the simulator as a training method that may be assessed by an evaluation of experts in the subject matter of the training [14-16,20]. The implementation of a new protocol and limited incidence of emergency resternotomies after cardiac surgery highlight the need to develop a high-fidelity training method that follows the expert consensus protocol for CPR and resternotomy for patients after cardiac surgery [2]. To facilitate medical staff training at our cardiothoracic surgery (CTS) department, we have developed a dedicated VR-based postcardiac surgery CPR simulation: Cardiopulmonary Resuscitation Virtual Reality Simulator (CPVR-sim). We designed a prospective study to assess the feasibility and to establish the face and content validity of CPVR-sim in a group of novices (eg, surgical residents, junior physicians, and nurse practitioners) and experts (eg, cardiothoracic surgeons and senior residents).

## Methods

### Simulator

The simulation was designed by a multidisciplinary team consisting of physicians, researchers, software developers, digital transformation experts, VR experts, and cardiothoracic surgeons from the CTS departments at Erasmus Medical Center (Rotterdam, the Netherlands), Zan Mitrev Clinic (Skopje, Republic of North Macedonia), and Distant Point LTD (Skopje, Republic of North Macedonia). Unreal Engine (Epic Games, Cary, North Carolina) software was used for software development. An Oculus Quest 2 (Oculus, Irvine, California) head-mounted display (HMD), in combination with two VR controllers and a high-performance laptop (MSI, New Taipei City, Taiwan), was used to run the CPVR-sim.

To study the feasibility of the CPVR-sim, we developed an immersive VR simulation resembling two CPR scenarios (both shockable and nonshockable cardiac arrest scenarios) after cardiac surgery, based on fictive patient cases (Multimedia Appendix 1). The patient scenarios recreated in the simulation were patients a few days after cardiac surgery through median sternotomy. These patients were found to be unresponsive on the surgical ward and determined to be in cardiac arrest and requiring CPR. In the first scenario, the cardiac arrest was caused by cardiac tamponade leading to pulseless electrical activity (PEA) where a resternotomy had to be performed to obtain the return of spontaneous circulation. The second scenario combined PEA and VF, and participants had to perform multiple actions, including external defibrillation, resternotomy, internal defibrillation, internal heart massage, and intracardiac medication administration.

**Figure 1:**
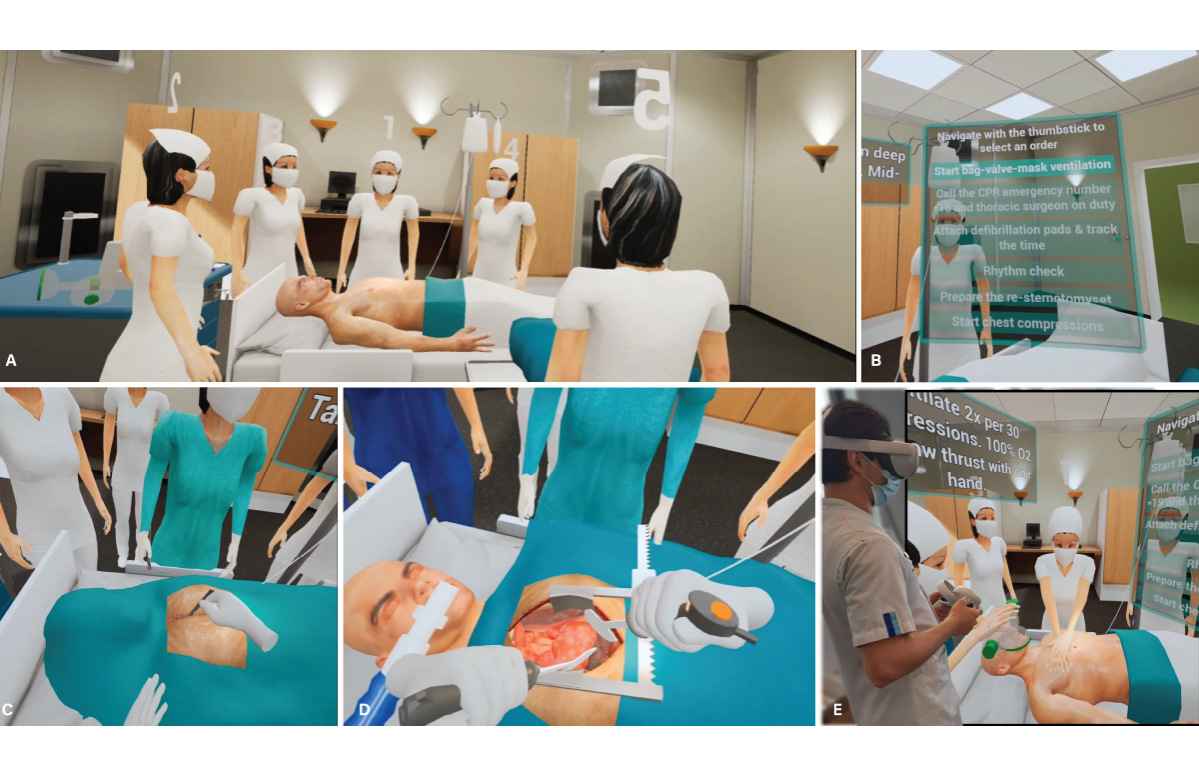
Screen captures of the Cardiopulmonary Resuscitation Virtual Reality Simulator (CPVR-sim) showing an overview with five virtual nurses in a patient room (A), the main menu (B), opening the incision with a virtual scalpel (C), performing the internal defibrillation (D), and a participant performing the simulation wearing the head-mounted display, with an in-screen screen capture of the CPVR-sim (E).

Before running the simulation, each participant was given a short briefing on the scenario, how to use the VR HMD, and how to interact with the controls and software to perform the CPVR-sim. When the simulation started, the user of the CPVR-sim was placed as a team leader of the CPR team. The team leader was able to assign tasks to the other participants in the simulation or was able to execute several tasks themself to manage the cardiac arrest situation. Figure 1 shows multiple screen captures of the team leader’s view during the simulation. The team leader instructed the virtual colleagues by choosing between different menu options (Figure 1B) with the joystick on the controller. Additionally, a participant wearing the HMD and performing the simulation is shown (Figure 1D). The menu options were shuffled each time the simulation started, so the user did not know the order of the menu options beforehand. When the correct command was given, it was followed by visual and auditory feedback of the instruction. This means, for example, that when “Start Chest Compressions” was chosen at the correct moment, the virtual nurse confirmed the instruction and started chest compressions.

### Study Participants

All participants work at the Erasmus MC in the Cardiothoracic Surgery Department as staff cardiothoracic surgeon, physician (including trainees in CTS), nurse practitioner, or medical student. To assess the content validity of the PEA scenario, participants were assigned to the novice or expert group. Staff cardiothoracic surgeons and certified CPR training instructors were categorized as expert, while the remaining participants were classed as novices (eg, junior physicians, nurse practitioners, surgical residents, and medical students). All participants completed written consent forms for their participation in this study. The research protocol was approved by the Erasmus Medical Center Medical Ethical Review Committee (MEC-2020-0989).

### Questionnaire

To assess participant characteristics, face validity, and content validity, a questionnaire was developed, which included experience with emergency resternotomy, gaming, and VR, among other things. Subsequent questions were scored on a five-point Likert scale, ranging from 1 (fully disagree) to 5 (fully agree). The Likert scale questions were divided into the following categories: usefulness, satisfaction, ease of use [21], effectiveness, and immersiveness [12,22-24], as described in previous studies. Finally, the last part of the questionnaire consisted of open questions to assess the advantages and disadvantages of the simulation. The questionnaire can be found in File S1 in Multimedia Appendix 2. To determine face validity, we used questionnaire results on the ease of use, effectiveness, and immersiveness of all participants. To assess content validity, we looked at the results from the expert group that performed the PEA scenario regarding usefulness and satisfaction.

### Statistical Analysis

Statistical analysis was performed using IBM SPSS Statistics for Windows, version 26.0 (IBM Corp). The chi-square test was used to perform statistical analyses of categorical data such as the participant characteristics. Continuous data are presented as medians with IQRs, and categorical data, including Likert scales, are presented as percentages.

## Results

### Participant Characteristics

All 41 participants performed the simulation and completed the questionnaire. Participants were divided into an expert and novice group to assess content and face validity of the PEA scenario. A total of 15 experts (staff cardiothoracic surgeons and certified CPR instructors) and 15 novices (physicians, residents, nurse practitioners, and medical students) were included in the PEA scenario. The median age of the expert group was 43 (IQR 38-55.5) years and of the novice group 30 (IQR 30-42.5) years (*P*<.001). Furthermore, the median work experience in CTS was 17 (IQR 9.5-26.5) years in the expert group and 1 (IQR 0.5-4.5) year in the novice group (*P*<.001). The participant characteristics are shown in Table 1.

**Table 1:** Participant characteristics.

Characteristic		PEA^a^ scenario, n (%)		Combined scenario (PEA + VF^b^), n (%)	Total (n=41), n (%)
		Experts (n=15)	Novices (n=15)	Experts + novices (n=11)	
**Sex**					
	Male	12 (80)	10 (67)	5 (45)	27 (66)
	Female	3 (20)	5 (33)	6 (55)	14 (34)
**Profession**					
	Cardiothoracic surgeon	13 (87)	1 (7)	0 (0)	14 (34)
	CTS^c^ resident	1 (7)	4 (27)	0 (0)	5 (12)
	CTS junior physician	0 (0)	6 (40)	6 (55)	12 (29)
	CTS nurse practitioner	1 (7)	4 (27)	1 (9)	6 (15)
	CTS medical student	0 (0)	0 (0)	4 (36)	4 (10)
**Experience with post–cardiac surgery CPR^d^**					
	No experience	0 (0)	1 (7)	5 (45)	6 (15)
	1-5 times	1 (7)	8 (53)	4 (36)	13 (32)
	6-10 times	1 (7)	4 (27)	1 (9)	6 (15)
	>10 times	13 (87)	2 (13)	1 (9)	16 (39)
**Experience with emergency resternotomy**					
	No experience	0 (0)	5 (33)	6 (55)	11 (27)
	1-5 times	4 (27)	9 (60)	4 (36)	17 (41)
	6-10 times	1 (7)	1 (7)	1 (9)	3 (7)
	>10 times	10 (67)	0 (0)	0 (0)	10 (24)
**Experience with gaming console**					
	Never used a gaming console	2 (13)	2 (13)	1 (9)	5 (12)
	Few times before	12 (80)	10 (67)	7 (64)	29 (71)
	Regular basis	1 (7)	3 (20)	3 (27)	7 (17)
**Experience with VR^e^**					
	Never had a VR experience	4 (27)	5 (33)	4 (36)	13 (32)
	Few times before	8 (53)	7 (47)	7 (64)	22 (54)
	Regular basis	3 (20)	2 (13)	0 (0)	5 (12)
	VR expert	0 (0)	1 (7)	0 (0)	1 (2)
**Experience with simulation training**					
	Never had simulation training	5 (33)	1 (7)	1 (9)	7 (17)
	Multiple times	8 (53)	14 (93)	9 (82)	31 (76)
	Certified trainer	2 (13)	0 (0)	1 (9)	3 (7)
**Experience with digital training**					
	Never had digital training	5 (33)	7 (47)	2 (18)	14 (34)
	Few times before	8 (53)	5 (33)	5 (45)	18 (44)
	Multiple times before	2 (13)	3 (20)	4 (36)	9 (22)

^a^PEA: pulseless electrical activity.

^b^VF: ventricular fibrillation.

^c^CTS: cardiothoracic surgery.

^d^CPR: cardiopulmonary resuscitation.

^e^VR: virtual reality.

### Questionnaires

**Figure 2:**
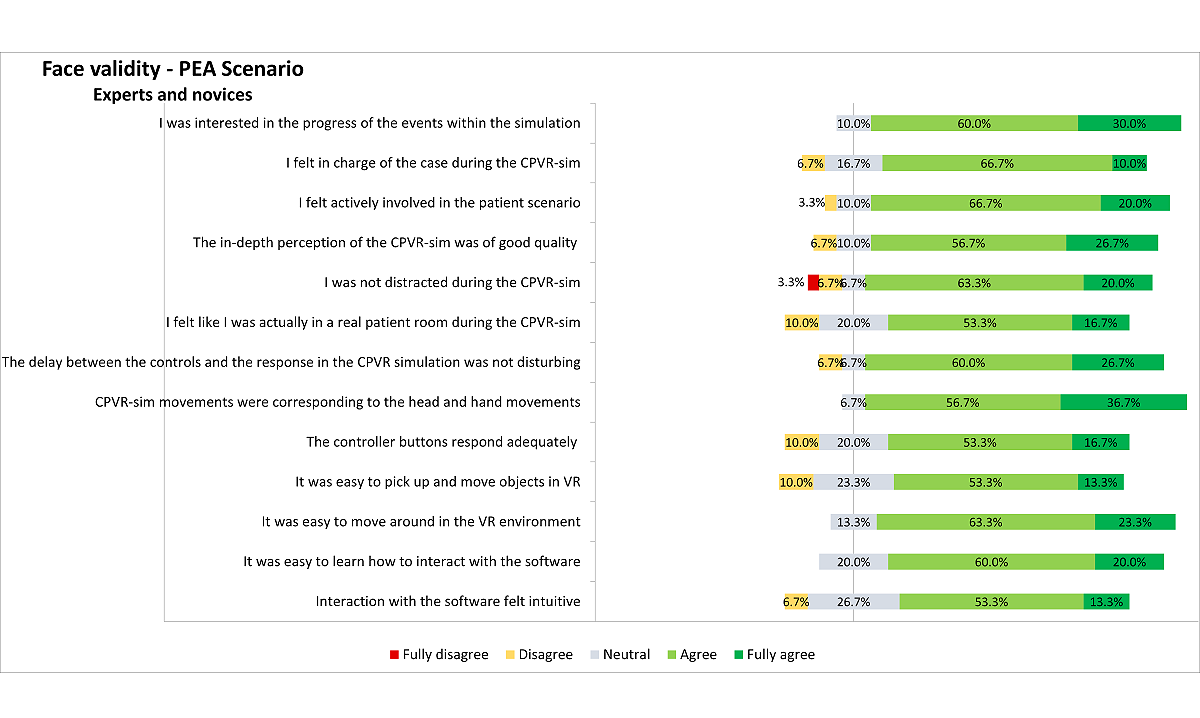
Representation of the results on face validity–related questionnaires assessed from all (expert and novice) participants on the PEA scenario. Inconsistencies in the sum of percentages is due to the rounding of the percentages. CPVR-sim: Cardiopulmonary Resuscitation Virtual Reality Simulator; PEA: pulseless electrical activity; VR: virtual reality.

**Figure 3:**
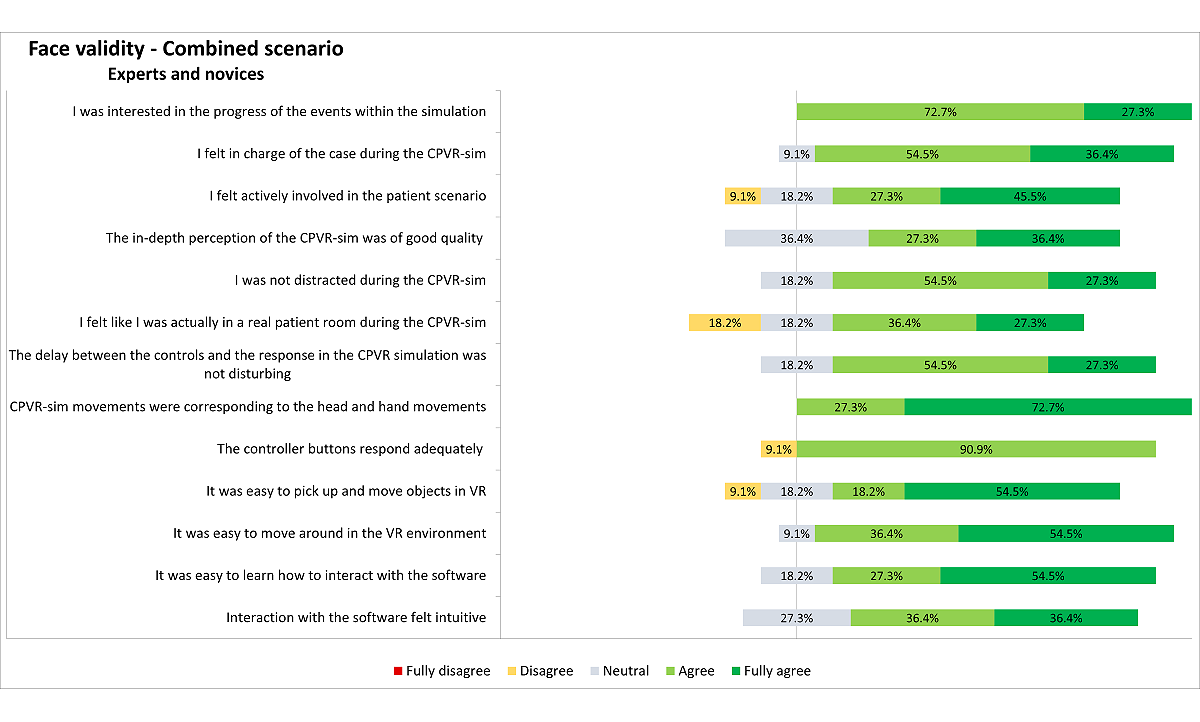
Representation of the results on face validity–related questionnaires assessed from all (expert and novice) participants on the combined scenario. Inconsistencies in the sum of percentages is due to the rounding of the percentages. CPVR-sim: Cardiopulmonary Resuscitation Virtual Reality Simulator; VR: virtual reality.

The face validity of both scenarios was assessed separately by analyzing the ease of use, effectiveness, and immersiveness questions in the questionnaires. The results of the PEA scenario are displayed in Figure 2. Most participants in the PEA scenario (n=26, 87%) felt actively involved, and 23 (77%) participants felt in charge of the situation, suggesting a predominant positive opinion regarding the face validity in both groups. The simulation software responded adequately and did not lag according to 21 (70%) of the participants, and 24 (80%) of the participants reported that it was easy to learn how to interact with the software. Notably, 12 (80%) of the novices in the PEA scenario said they learned a lot from the simulation, whereas only 7 (47%) experts reported the same. The results of the combined scenario are displayed in Figure 3. Additionally, 10 (91%) of the participants stated that it was easy to move around in the VR environment, and the same amount of people reported that the controller buttons responded adequately.

**Figure 4:**
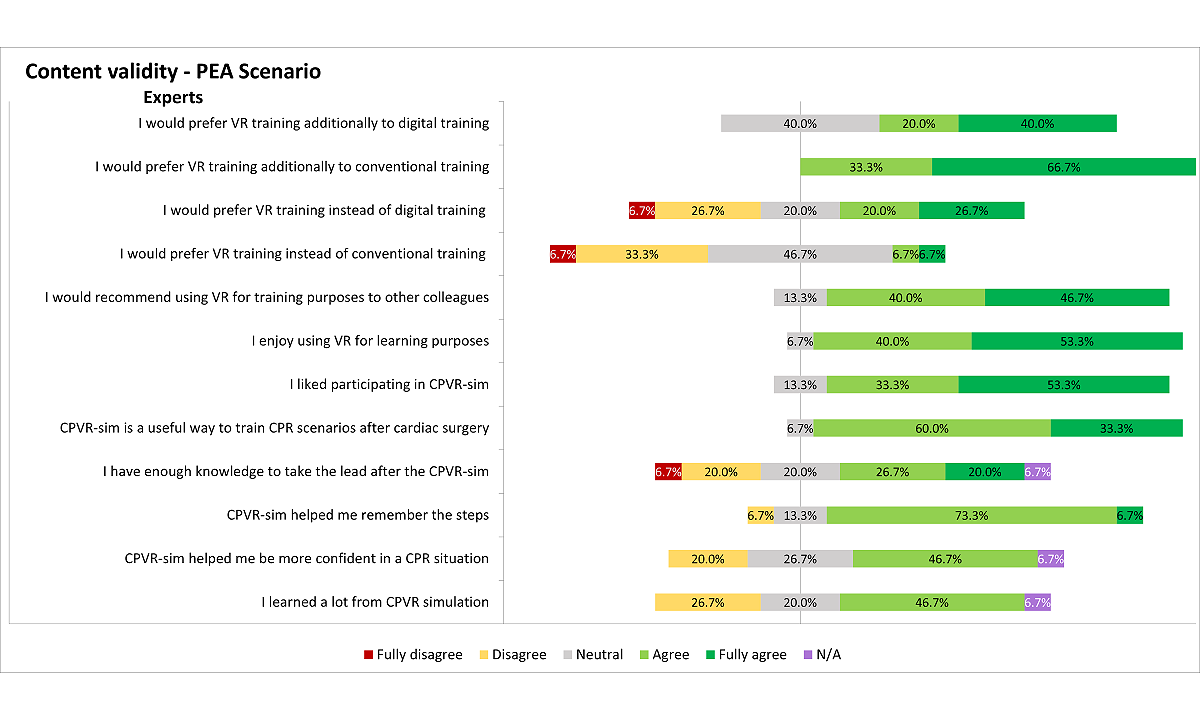
Representation of the results on content validity–based questionnaires of the PEA scenario, assessed from the expert participants. Inconsistencies in the sum of percentages is due to the rounding of the percentages. CPR: cardiopulmonary resuscitation; CPVR-sim: Cardiopulmonary Resuscitation Virtual Reality Simulator; N/A: not applicable; PEA: pulseless electrical activity; VR: virtual reality.

Subsequently, the content validity was assessed by analyzing the satisfaction and usefulness outcomes of the questionnaire of the expert group (n=15) who performed the PEA scenario (Figure 4).

All expert participants in the PEA scenario (n=15, 100%) agreed that this VR training method is useful as a supplement to conventional training methods, and 9 (60%) participants agreed it was useful as a supplement to digital training. Notably, only 2 (14%) expert participants would prefer VR training instead of conventional training; however, 7 (47%) expert participants would prefer VR training instead of digital training. Conversely, 13 of 15 (87%) expert participants would recommend VR training to other colleagues, and most participants (n=14, 93%) reported that the CPVR-sim was a useful method to train infrequently occurring CPR cases after cardiac surgery (File S2 in Multimedia Appendix 2).

Finally, participants were asked for general advantages and disadvantages of the CPVR-sim. The most commonly reported advantages were the broad applicability of VR simulation in various CPR scenarios; the possibility of repetitive, personal, and quick practice sessions without being restricted by logistical challenges; and that the CPVR-sim is a beneficial method for step-by-step sequence training. Additionally, many participants felt it was a fun way of learning. The most important disadvantages of the current CPVR-sim version were the limited freedom of decision-making, lack of team training and interaction with a team, and the absence in the CPVR-sim of the pressure and hectic environment during such an emergency situation, which occasionally made it feel artificial. Results of the face and content validity questionnaire can be found in File S2 in Multimedia Appendix 2, and a complete overview of the advantages and disadvantages filled in by the participants can be found in File S3 in Multimedia Appendix 2.

## Discussion

### Principal Results

In this prospective study, we have designed and evaluated a VR simulation training platform with two different scenarios for post–cardiac surgery emergencies requiring CPR. To the best of our knowledge, this is the first time that such a VR simulation platform has been developed explicitly for use in a cardiac arrest scenario after cardiac surgery. Although future refinements of this concept are inevitable, we believe that the CPVR-sim will be a successful method that will help to overcome difficult challenges, including the infrequent incidence of resternotomy after cardiac surgery, accessibility, and costs of clinical training [25]. We observed that the expert and novice opinions were generally positive regarding the face validity and content validity. A significant majority of participants from both groups felt that VR simulations are a useful (supplementary) training method, as well as a high likelihood that they would recommend VR simulation training to other colleagues. Furthermore, in the CPVR-sim scenarios, the trainee was more actively involved in experiencing the virtual patient case, as compared with conventional digital training, listening to a presentation, or reading a protocol. This active involvement could be due to the elaborate simulation and multiple actions the user has to perform. This immersive and realistic VR environment facilitates memorizing stepwise procedures more efficiently [25]. Additionally, our results showed that frequent practice and increased exposure in the CPVR-sim is valuable since it refreshes the knowledge and gives the clinician more confidence in taking the lead in future situations, which is in line with previous studies [8,9,12,13]. This is especially important in infrequent CPR cases with emergency resternotomy, which occurs only a few times per year.

Another important feature of this VR training is the improved accessibility since the only requirements are an HMD and a computer, and there is no need to arrange a physical session. VR training has higher initial costs (eg, simulation development and purchase costs of the VR hardware) than conventional training. However, the increased accessibility of VR training results in more trainees being reached, spreading these initial costs over a larger group, compared with the relatively linear cost per trainee for conventional training. Therefore, the average cost per trainee would likely be lower in the long term for VR training than conventional training [26]. Moreover, purchasing and using VR hardware adds a new dimension and armature in training possibilities and other applications (ie, surgical planning) of a department, which can also lead to cost-efficiency. Finally, VR training facilitates the implementation of the new CPR protocol, enabling training for experts and novices alike who are not yet acquainted with the new protocol.

### Limitations

In this simulation, only individual training was possible. It would be desirable to make the simulation available for multiple users at a time, enabling real-time interaction between team members [11]. By making the simulation available for multiple users at the same time, nontechnical skills such as communication and leadership can be trained with the team, which is important in CPR situations [27]. This would also enable learning from other trainees’ mistakes. Multiplayer settings would additionally enable the trainee to view the CPR situation from different viewpoints and the possibility to review their own performance from an alternative perspective [25]. Another shortcoming of the CPVR-sim is that the simulation requires at least five different buttons to be pressed, which can be confusing for the trainee. The simulation would become more realistic and interactive when voice controls and haptic feedback such as hand or even body tracking are implemented to perform the actions within the simulation, instead of using the controllers as input in the simulation [28]. However, implementing voice control can be computationally and algorithmically challenging, as similar information can be said using a variety of different phraseology, and further research should be performed on the best interaction method within the VR environment [25-28].

Finally, a shortcoming in this simulation was the lack of pressure felt by participants and the absence of a hectic environment, characteristic of such an emergency situation. The virtual nurses stood still and walked calmly, and there was a lack of background noise. This could be improved in future development by adding stress components such as sounds or extra persons who are panicking [20]. Making the simulation more resembling the real-life situation might improve the success of VR training [25]. However, further research is needed to ensure such stress components do not compromise the educational value of the CPVR-sim.

### Future Perspective

The most crucial next step in improving the simulation and increasing educational value is to extend the CPVR-sim with different roles in the CPR simulation, for example, for nurses. With this functionality, multiplayer scenarios will become possible, and a team can train together at the same time, ultimately creating a more realistic environment that will translate more directly into clinical practice. Furthermore, the simulation can be improved by adding more scenarios, asystole, and external pacing, for example.

In this study, we only assessed the content validity of the PEA scenario, but in future studies, the content must be validated for all different scenarios of the CPVR-sim. These face and content validity results support the use of the simulator as a training tool, but they are subjective measures of validity, and it is imperative to validate the simulation objectively. This can be perceived by determining the construct validity, concurrent validity, and predictive validity of the CPVR-sim [14,15,20]. Construct validity in a simulation is defined as the ability to distinguish objectively between different levels of experience [14,15,20]. In future research, this could be determined by testing a large number of users with various levels of experience in CPR and emergency resternotomy cases after cardiac surgery. Concurrent validity can determine the correlation between the VR simulation and existing evaluation tools [15,20]. Moreover, predictive validity is an even more powerful evidence method, which can be assessed by comparing the outcomes of the simulation with an established assessment method to assess the skills [15,20]. In further research, predictive validity could be determined by comparing the clinical staff’s skills in a real-life simulation setting and CPVR-sim. These skills could be obtained by a structured skills assessment of both the skills in real life and within the VR simulation, determined by blinded experienced CPR trainers.

### Conclusion

We have developed a proof-of-concept VR simulation of two CPR scenarios after cardiac surgery, which is immersive and useful, as stated by the expert and novice participants. Additional research is needed to further develop and validate the simulation platform, including multiple possible clinical scenarios; voice control; multiuser possibilities; and assessing the construct, concurrent, and predictive validity. However, we made a first step toward a CTS VR training platform, including multiple realistic and repetitive simulation training for the CTS department by proving the face validity and content validity of the CPVR-sim.

## Appendix

Multimedia Appendix 1 Supplementary video of the cardiopulmonary resuscitation virtual reality simulator.

Multimedia Appendix 2 Supplementary file.

## Abbreviations

CPR: cardiopulmonary resuscitation
CPVR-sim: Cardiopulmonary Resuscitation Virtual Reality Simulator
CTS: cardiothoracic surgery
HMD: head-mounted display
PEA: pulseless electrical activity
VF: ventricular fibrillation
VR: virtual reality
