# Supplementary material for: Virtual Reality Simulation Training for Cardiopulmonary Resuscitation After Cardiac Surgery: Face and Content Validity Study
Source: JMIR Serious Games. 2022 Mar 2;10(1):e30456. doi: 10.2196/30456 (PMC8928050; doi:10.2196/30456)
Supplement: Multimedia Appendix 2 [file games_v10i1e30456_app2.docx]

**Supplementary file S1**

**User experience questionnaire “CPVR-Sim”**

Male/female Age: __________ Profession: ________________________________

1. How many years of work experience do you have in thoracic surgery working as a physician or nurse practitioner? ________________________________________________________
2. How many post-cardiac surgery resuscitations did you participate in?

- I have never participated in a CPR situation
- 1-5 times
- 5-10 times
- More than 10 times

1. How many emergency resternotomy procedures did you participate in?

- I have never participated in an emergency resternotomy situation
- 1-5 times
- 5-10 times
- More than 10 times

1. Do you have experience with gaming consoles (e.g. computer gaming, xbox, playstation)?

- I have never used a gaming console
- I have used a gaming console a few times before
- I am gaming on a regular basis (at least once a month)

1. How often do you use VR hardware/software (e.g. VR gaming, simulations, consoles, entertainment etc.)?

- I have never had a VR experience until today
- I have used VR a few times before
- I am experienced and use VR on a regular basis (at least once a month)
- I am an VR expert (have a VR console and applications myself)

1. Do you have experience with physical simulation trainings (e.g CPR or ALS trainings)?

- I have never had simulation training before
- I have had simulation trainings multiple times before
- I am a certified simulation trainer

1. Do you have experience with digital training (e.g. e-learning or serious games)?

- I have never had such training before
- I have had a digital training a few times before
- I have had digital trainings multiple times before

1. Do you have experience with a simulation training in VR?

- Yes
- No

|  |  | 1. Fully disagree | 2. Disagree | 3. Neutral | 4. Agree | 5. Fully agree | N/A |
| --- | --- | --- | --- | --- | --- | --- | --- |
|  | **Usefullness** |  |  |  |  |  |  |
| 1 | I learned a lot from the CPR protocol after cardiac surgery in the CPVR simulation |  |  |  |  |  |  |
| 2 | The CPVR simulation helped me being more confident in taking the lead in a future CPR situation |  |  |  |  |  |  |
| 3 | The CPVR simulation helped me remember the steps in a CPR and resternotomy situation |  |  |  |  |  |  |
| 4 | After the CPVR simulation, I have enough knowledge to take the lead in a future CPR situation after cardiac surgery |  |  |  |  |  |  |
| 5 | CPVR simulation is a useful way to train CPR scenarios after cardiac surgery |  |  |  |  |  |  |
|  | **Satisfaction** |  |  |  |  |  |  |
| 6 | I liked participating in the CPVR simulation |  |  |  |  |  |  |
| 7 | I enjoy to use VR for learning purposes |  |  |  |  |  |  |
| 8 | I would recommend using VR for training purposes to other colleagues |  |  |  |  |  |  |
| 9 | I would prefer VR training instead of conventional training (in-classroom training with PowerPoint and a simulation with multiple participants) |  |  |  |  |  |  |
| 10 | I would prefer VR training instead of digital training (e.g. e-learning or serious game) |  |  |  |  |  |  |
| 11 | I would prefer VR training additionally to conventional training (in-classroom training with PowerPoint and a simulation with multiple participants)? |  |  |  |  |  |  |
| 11 | I would prefer VR training additionally to digital training (e.g. e-learning or serious game) |  |  |  |  |  |  |
|  | **Ease of use** |  |  |  |  |  |  |
| 11 | The interaction with the CPVR software felt intuitive |  |  |  |  |  |  |
| 12 | It was easy to learn how to interact with the software |  |  |  |  |  |  |
| 13 | It was easy to move around in the VR environment |  |  |  |  |  |  |
| 14 | It was easy to pick up and move objects in the VR environment |  |  |  |  |  |  |
|  |  | 1. Fully disagree | 2. Disagree | 3. Neutral | 4. Agree | 5. Fully agree | N/A |
|  | **Effectiveness** |  |  |  |  |  |  |
| 15 | The CPVR simulation is responding adequately and is not lacking when using the buttons on the controllers |  |  |  |  |  |  |
| 16 | When moving the head and hands with the HMD, the CPVR simulation moved corresponding to the movements |  |  |  |  |  |  |
| 17 | There delay between the (movements of the) controls and the response in the CPVR simulation was not disturbing |  |  |  |  |  |  |
|  | **Immersiveness** |  |  |  |  |  |  |
| 18 | I felt like I was actually in a real patient room during the CPVR simulation |  |  |  |  |  |  |
| 19 | I was not distracted during the CPVR simulation |  |  |  |  |  |  |
| 20 | The in-depth perception of the CPVR simulation was of good quality |  |  |  |  |  |  |
| 21 | I felt actively involved in the patient scenario of the CPVR simulation |  |  |  |  |  |  |
| 22 | I felt in charge of the case during the CPVR simulation |  |  |  |  |  |  |
| 23 | Communication with the colleagues in the CPVR simulation felt natural |  |  |  |  |  |  |
| 24 | I was interested in the progress of the events within the simulation |  |  |  |  |  |  |

Write down the advantages and disadvantages of the CPVR simulation for CPR and resternotomy training, rank them in order of importance, from most important (1) to least important (3).

**Advantages:**

1.

2.

3.

**Disadvantages:**

1.

2.

3.

Do you have any comments or did you miss something in the simulation?

__________________________________________________________________

__________________________________________________________________

__________________________________________________________________

__________________________________________________________________

Thank you for participating!

**Supplementary file S2**

**Results on the questionnaires of the Face validity and content validity of all participants**

**Table 1: Face Validity**

|  | **PEA Scenario** | | | | **Combined scenario** | | **Total** | |
| --- | --- | --- | --- | --- | --- | --- | --- | --- |
|  | Experts | | Novices | | Experts + novices | |  |  |
|  | (n=15) | | (n=15) | | (n=11) | | (n=41) | |
|  | Median | IQR | Median | IQR | Median | IQR | Median | IQR |
| **Ease of use** |  |  |  |  |  |  |  |  |
| Q11.2:The interaction with the CPVR software felt intuitive | 4 | 1 | 4 | 1 | 4 | 1,5 | 4 | 1 |
| Q12:It was easy to learn how to interact with the software | 4 | 0,5 | 4 | 1 | 5 | 1 | 4 | 1 |
| Q13:It was easy to move around in the VR environment | 4 | 0 | 4 | 1 | 5 | 1 | 4 | 1 |
| Q14:It was easy to pick up and move objects in the VR environment | 4 | 0 | 3 | 1 | 5 | 1,5 | 4 | 1 |
| **Effectiveness** |  |  |  |  |  |  |  |  |
| Q15:The CPVR simulation is responding adequately and is not lacking when using the buttons on the controllers | 4 | 0 | 4 | 1 | 4 | 0 | 4 | 0 |
| Q16: Giving voice commands is functioning adequately in the CPVR simulation | 4 | 0,25 | 3 | 1,5 | 4 | 0,5 | 4 | 1 |
| Q17:When moving the head and hands with the HMD, the CPVR simulation moved corresponding to the movements | 4 | 4,5 | 4 | 1 | 5 | 0,5 | 4 | 1 |
| Q18:There delay between the (movements of the) controls and the response in the CPVR simulation was not disturbing | 4 | 0 | 4 | 1 | 4 | 0,5 | 4 | 1 |
| **Immersiveness** |  |  |  |  |  |  |  |  |
| Q18.1:I felt like I was actually in a real patient room during the CPVR simulation | 4 | 0,5 | 4 | 1,5 | 4 | 1,5 | 4 | 1 |
| Q20:I was not distracted during the CPVR simulation | 4 | 0 | 4 | 1 | 4 | 0,5 | 4 | 0 |
| Q21:The in-depth perception of the CPVR simulation was of good quality | 4 | 0 | 4 | 1 | 4 | 2 | 4 | 1 |
| Q22:I felt actively involved in the patient scenario of the CPVR simulation | 4 | 0 | 4 | 0 | 4 | 1,5 | 4 | 1 |
| Q23: I felt in charge of the case during the CPVR simulation | 4 | 0 | 4 | 0,5 | 4 | 1 | 4 | 0 |
| Q24:Communication with the colleagues in the CPVR simulation felt natural | 3 | 1 | 2 | 1,75 | 3 | 1 | 3 | 2 |
| Q25:I was interested in the progress of the events within the simulation | 4 | 1 | 4 | 0,5 | 4 | 0,5 | 4 | 1 |

| **Table 2: Content Validity of the PEA scenario** | |  |  |  |  |  |  |
| --- | --- | --- | --- | --- | --- | --- | --- |
|  | Experts | | Novices | | Total | |  |
|  | (n = 15) | | (n=15) | | (n=30) | |  |
|  | Median | IQR | Median | IQR | Median | IQR | P value* |
| **Usefullness** |  |  |  |  |  |  |  |
| Q1: I learned a lot from the CPR protocol after cardiac surgery in the CPVR simulation | 3.5 | 1.75 | 4 | 0 | 4 | 1 | 0.123 |
| Q2:The CPVR simulation helped me being more confident in taking the lead in a future CPR situation | 3.5 | 1 | 4 | 0 | 4 | 1 | 0.05 |
| Q3:The CPVR simulation helped me remember the steps in a CPR and resternotomy situation | 4 | 0 | 4 | 0 | 4 | 0 | 0.172 |
| Q4: After the CPVR simulation, I have enough knowledge to take the lead in a future CPR situation after cardiac surgery | 3.5 | 1.75 | 3 | 2 | 3 | 2 | 0.769 |
| Q5:CPVR simulation is a useful way to train CPR scenarios after cardiac surgery | 4 | 1 | 4 | 1 | 4 | 1 | 0.567 |
| **Satisfaction** |  |  |  |  |  |  |  |
| Q6:I liked participating in the CPVR simulation | 5 | 1 | 5 | 0 | 5 | 1 | 0.233 |
| Q7:I enjoy to use VR for learning purposes | 5 | 1 | 5 | 0.5 | 5 | 1 | 0.325 |
| Q8: I would recommend using VR for training purposes to other colleagues | 4 | 1 | 5 | 0.5 | 5 | 1 | 0.285 |
| Q9: I would prefer VR training instead of conventional training (in-classroom training with PowerPoint and a simulation with multiple participants) | 3 | 1 | 3 | 0 | 3 | 1 | 0.775 |
| Q10: I would prefer VR training instead of digital training (e.g. e-learning or serious game) | 3 | 2.5 | 4 | 2 | 4 | 2.75 | 0.436 |
| Q11:I would prefer VR training additionally to conventional training (in-classroom training with PowerPoint and a simulation with multiple participants) | 5 | 1 | 5 | 1 | 5 | 1 | 0.838 |
| Q12:I would prefer VR training additionally to digital training (e.g. e-learning or serious game) | 4 | 2 | 5 | 1 | 4 | 2 | 0.367 |
| * Mann-Whitney U test |  |  |  |  |  |  |  |

**Supplementary file S3**

Advantages and disadvantages of the “CPVR-sim” from the questionnaire by the participants

**Participant 2:**

Advantages:

1. It can be applied on all situations

2. You need little supplies such as a simulation doll

Disadvantages:

1. It can be unnaturally

2. Limited options because of the menu, using voice controlled orders would be more realistic

3. You don’t train with your own team

**Participant 3:**

Advantages:

1. Easy accessible

2. Frequent practice

Disadvantages:

1. Does not mimic the pressure of real life training with colleagues

2. Fixed steps towards the goal, while normally several steps happen simultaneously

3. Does not practice technique, CPR or resternotomy

**Participant 4:**

Advantages:

1. "Hands on" training of real situation

2. Frequent practice without need for extra material of persons

3. Easy actions

Disadvantages:

1. Actions before resternotomy not fully according to reality

2. Missing hapting feedback during resternotomy

**Participant 5:**

Advantages:

1. Immersive experience of a situation that occurs infrequently

2. Training for all new residents

3. Personal and quick practice session possible, instead of arranging a physical lesson

Disadvantages:

1. In real life various procedures happen at once, instead of after each other (as in this simulation

2. Hand tracking instead of controllers would feel more natural

3. Only 1 resident/ nurse can participate in this setting

**Participant 6:**

Advantages:

1. Realistic

2. Practising resternotomy

Disadvantages:

1. Less intuitive testing of decision making

**Participant 9:**

Advantages:

1. Good way to test the order and actions

2. Easier available and accessible than "real life" training

Disadvantages:

1. Not 100% translatable to real life, it is different than real life

**Participant 10:**

Advantages:

1. Much better visualisation than in ppt or in a simulation with a doll

2. Step by step clear visualisation

Disadvantages:

1. It stays virtual, so the hands-on feeling misses

2. Scenario is very quiet, more than in a real resuscitation scenario

**Participant 11:**

Advantages:

1. Real-life training anytime, not bounded to logistic challenges

2. Repetitive training, low cost on the long run

Disadvantages: none

**Participant 13:**

Advantages:

1. Time efficient

2. Visualization ensures better memorization

Disadvantages:

1. No real time team interaction like in classroom trainings

2. No learning from mistakes of other participants

**Participant 14:**

Advantages:

1. Sequence training

2. Unlimited training/ repetitive possibilities

3. Notice Ideal setting as a reference

Disadvantages:

1. Not practical tools (hand)

2. Missing tactile feedback

3. Non-natural movements

**Participant 15:**

Advantages:

1. Step by step going through the protocol

2. Pretty views/ visualisation

3. Responds well on given commands

Disadvantages:

1. Only one order can be given at a time, in real life you give these orders in one breath

2. Head mounted display needs to be attached very tightly, not much space for glasses

3. Before participating in the simulation you need to be aware of all the buttons to be used

**Participant 17:**

Advantages:

1. Very helpful in understanding and learning the CPR protocol, gives me more confidence

Disadvantages:

1. Learning curve with the controllers, but when I was used to it it was very easy

**Participant 18:**

Advantages:

1. Fast practical method of going through the protocol

2. Experience the situation instead of reading it

Disadvantages:

1. One sided in roles, only the surgeon role can be executed

2. Not yet with actual voice controlling, but with the menu

**Participant 20:**

Advantages:

1. Learning of young colleagues who never experienced such scenario

2. Different simulation possibilities

3. Refresh your mind

Disadvantages:

1. Still in simulation

2. In practice it is always different, more hectic and more people

**Participant 21:**

Advantages:

1. Realistic simulation with important practical aspects, similar to reality

2. Immersive and very nice to rehearse resternotomy steps in VR

3. More confident for future situations

Disadvantages:

1. Single user

2. PEA only one single scenario

**Participant 23:**

Advantages:

1. Very nice substitution to CPR training

2. Makes a good platform for future

3. Much more interactive

Disadvantages:

1. A bit small on a proper environment

2. Use of medications could be very useful

**Participant 24:**

Advantages:

1. Additional trainings module as an extra next to theory and practice

2. More scenarios can be built in

3. Schematic training of steps is well possible

Disadvantages:

1. Gives a too simplistic view of reality

2. Only one scenario is possible during training

3. Hiccup’s in the software

**Participant 25:**

Advantages:

1. Widely accessible

2. Intuitive

3. Fun!

Disadvantages: none

**Participant 26:**

Advantages:

1. Very realistic/immersive

2. Possibility to simulate a wide variety of situations/scenario's

3. Ability to train anytime, anywhere and thereby increasing exposure

Disadvantages:

1. Suggestive instructions/dialogue, little freedom in decision making

**Participant 27:**

Advantages:

1. Multiple scenarios possible

2. Easy repeated training possible

3. Using for multiple players together

Disadvantages:

1. More choices possible but only one is correct in this training scenario

2. Only one person in action

3. Need for instructions before/during usage

**Participant 28:**

Advantages:

1. Getting familiar with the steps in a post cardiac surgery CPR setting

2. Open the resternotomy set

Disadvantages: none

**Participant 30:**

Advantages: none

Disadvantages:

1.I had no experience with VR, so it felt quite unreal

**Participant 31:**

Advantages:

1.Feels real

Disadvantages:

1.Not very elaborate (yet)

**Participant 32:**

Advantages:

1.Good to practice the CPR simulation step by step in a non-real life situation

Disadvantages:

1.It was only a part of CPR situation. Beginning can be more elaborate (e.g. including quick look)

**Participant 33:**

Advantages:

1.Realistic, better to memorize

2.Available at anytime

3.Good practice skills

Disadvantages:

1.Can’t talk back to colleagues, need more interaction

**Participant 34:**

Advantages:

1.Good to acquire knowledge about the CPR protocol

2.Very playful and fun way of learning

Disadvantages:

1.Not realistic

2.Does not simulate the stress you can experience during real life CPR

**Participant 35:**

Advantages:

1.Availability (compared to real-life simulation)

2.Possibility of wide-range of scenarios and interaction

3.Fun

Disadvantages:

1.Current module: restricted to following the module and multiple choices

**Participant 36:**

Advantages:

1.Realistic environment where you can prepare/train for future scenarios

2.Easy to use interface of VR simulation

3.Case description is realistic/commonly encountered

Disadvantages:

1.No good/clear interaction with nurse (verbal)

2. No tactile feedback

**Participant 37:**

Advantages: none

Disadvantages: none

**Participant 38:**

Advantages:

1.More intuitive/impressive

2.Sense of involvement

3.Informative

Disadvantages:

1.Not very convenient to perform

2.Feels unreal

**Participant 39:**

Advantages:

1.More of a real life experience than other courses could offer

2.Makes me remember the steps/order of actions better

3.Makes me think actively about next action in surrounding and people present

Disadvantages:

1.Did not know all the answers due to not being familiar with the protocol

2.Actions are not performed in detail

3.VR lasses are a bit heavy and not glasses friendly

**Participant 40:**

Advantages:

1.It seems a life scenario and it is more impressive than a PowerPoint presentation

2.Can be repeated until you’ve perfected the scenario which you can apply in real life

3.It is a lot more interactive, and possibly more fun to remember

Disadvantages:

1.You practice in a setting without critical components e.g. fluids, blood and without vocal communication

2.Maybe very tiring if done for a long time

3.The pace may be too fast for it being applies instead of a PowerPoint, PowerPoint and CPVR seems like the best option

**Participant 41:**

Advantages:

1.The way of learning is towards truthful

2.Easy accessible to learn without supervision

Disadvantages:

1.It is still individual, not with more people at the same time
